# Role of Inflammation in Endophthalmitis

**DOI:** 10.1155/2012/196094

**Published:** 2012-09-02

**Authors:** J. L. Vallejo-Garcia, M. Asencio-Duran, N. Pastora-Salvador, P. Vinciguerra, M. R. Romano

**Affiliations:** ^1^Ophthalmology Department, Istituto Clinico Humanitas, Rozzano, Milan, Italy; ^2^Universitary Hospital La Paz, Madrid, Spain

## Abstract

Inflammation originating from infection of the vitreous cavity is called endophthalmitis. Attention has been focused on the epidemiologic, microbiologic reports, and treatment options; unfortunately, the role of the host immune reaction in the visual function damage is still not well understood. Endophthalmitis occurs most frequently after cataract surgery. In this paper we review the published literature regarding inflammatory mediators and apoptosis during the course of endophthalmitis. Toll-like receptors, cytokines, high-mobility group box 1 proteins, aB-crystallin and apoptosis have been studied during clinical and experimental cases of endophthalmitis. Further understanding of the host-immune reaction to vitreous infection is essential for the development of new therapies. The use of intravitreal antibiotics and corticosteroids, vitrectomy and systemic antibiotics for the preservation of visual function is still discouraging.

## 1. Role of Inflammation in Endophthalmitis 

Endophtalmitis is defined as inflammation originating from infection of the vitreous cavity. The specific features of the cellular damage that is created from the excessive immune response are still not well understood. The inflammatory cascade activated by the specific toxic effects of the pathogen ultimately determines the final anatomical and functional visual outcome. Treatments available to neutralize the infection and to diminish the inflammatory damage are intravitreal antibiotics, intravitreal corticosteroids and vitrectomy. Although systemic antibiotics did not demonstrate any added treatment benefit in the Endophthalmitis Vitrectomy Study (EVS) [1], systemic therapeutic agents are currently widely used as their intraocular penetration and spectrum range has significantly improved. It is not clear whether the most severe damage to the visual function is caused by the infectious process or by the host immune response. Endophthalmitis is classified according to its origin as exogenous (postsurgical, after penetrating trauma, or contiguous infection) and endogenous or metastatic. It is also classified according to its presentation as acute, if it occurs within 6 weeks of surgery, or chronic, more than 6 weeks following surgery. Endophthalmitis after cataract surgery is responsible for 90% of endophthalmitis cases [2]. The incidence of endophthalmitis after cataract surgery ranges between 0.087 and 0.265% [3, 4].

Bacteria are responsible for the majority of endophthalmitis cases and the prevalence is higher in tropical locations. Isolated outbreaks have also been reported due to contamination of ocular irrigation fluids during surgery [5, 6]. Positive cultures were obtained in 69% samples in the EVS and coagulase-negative staphylococci. Most frequent germs are coagulase-negative staphylococci, accounting for more than 50% of the positive cultures, followed by other gram-positive germs like *Streptococcus* spp. and *Staphylococcus aureus.* Gram-negative organisms were responsible for 6% of cases and 2 or more organisms were found in 2.4 to 4% of cases [1, 7]. 

The ability of bacteria to cause endophthalmitis is related to the bacterial load and to the virulence of the organism such as rapid replication in the eye and the production of toxins that produce inflammatory reaction and cellular necrosis. Gram-positive cell wall components such as peptidoglycan, lipoteichoic acid and capsular polysaccharide have intraocular proinflammatory properties even if the organisms themselves are inactive [8, 9]. Gram-negative cell walls contain lipopolysaccharides, which are also proinflammatory [10]. The production of different types of bacterial enzymes such as hemolysins, lipases, enterotoxins, proteases, collagenases and hyaluronidases damages the host tissue. In addition to bacterial growth and direct toxicity, excessive host inflammatory response is responsible for impaired visual outcome due to photoreceptor toxicity; as these cells do not replicate, it is essential to minimize the collateral damages caused by inflammation. Ocular tissue evolution has developed an immune-privileged microenvironment to suppress the destruction of its cells, as it is critical to preserve the integrity and functionality of retinal cells and the clarity of the visual axis. This requires the preservation of specific anatomic characteristics such as the blood-ocular barrier and soluble immunosuppressive factors. 

Suppressor immunity is expressed by the induction of antigen-specific efferent suppressor CD8 T cells and afferent suppressor CD4 T cells also known as T-reg cells [11]. The ocular microenvironment is rich in immunosuppressive molecules that influence the activity of immune cells, such as neuropeptides-like *α*-Melanocyte-stimulating hormone (*α*-MSH), vasoactive intestinal peptide (VIP) and somatostatin (SOM), the cytokines-like transforming growth factor beta-2 (TGF-*β*2), indoleamine 2,3-dioxygenase enzyme (IDO), prostaglandin E2, and surface expression of FasL to suppress the activation of Th1 cells [12]. The presence of migration inhibitory factor (MIF) in aqueous humor prevents natural-killer-cell activation [13]. They not only suppress endotoxin-induced inflammatory activity, but also induce an anti-inflammatory cytokine production by macrophages. Breakdown of these mechanisms that preserve immune privilege from inflammatory eye diseases such as endophthalmitis, uveitis and keratitis can result in destruction of host tissue and loss of vision. This breakdown can be triggered by both infectious and immune mechanisms, toll-like receptors (TLRs), cytokines are part of the initiation of the eye immune response. Direct cellular damage and apoptosis are the consequences of the bacterial attack and host immune reaction. 

## 2. Toll-Like Receptors 

TLRs are a family of receptors that recognizes microbial-associated molecular patterns from diverse organisms, including bacteria, viruses, fungi and parasites (Table 1) [14]. Activation of TLRs on immune cells by pathogens or their products initiates the innate response characterized by the expression of proinflammatory mediators and antimicrobial effector molecules, responsible for recruiting immune cells to the site of infection, mediating host inflammatory response to injury and stress and tissue repair [14, 15]. Initial inflammatory responses mediated by TLRs are required for host defense against invading pathogens. 

TLRs have been identified in many cells throughout the eye, including retinal pigment epithelial (RPE) cells, astrocytes, corneal epithelium, iris epithelium, retinal microglia and Muller cells [14, 16, 17]. The presence of TLRs in microglia and Muller glial cells constitute an important feature in the recognition and initiation of the innate response to live pathogens and other microbial products such as lipopolysaccharids, other lipoproteins, peptiglicans, hemolysins, phospholipases, enterotoxins and proteases [14, 17].

Experimental models of TLR attenuated animals have demonstrated that the damage induced is lower than in normal TLR models. Novosad et al. [15] demonstrated that TLR2 deficient mice model of endophthalmitis resulted in decreased intraocular proinflammatory cytokine/chemokine levels and altered recruitment of inflammatory cells into the eye, resulting in less intraocular inflammation and preservation of retinal architecture, and a slightly greater degree of retinal function. Kumar et al. [18] demonstrated that a TLR2 ligand, Pam3Cys, injected intravitreally previously to an *S. aureus* endophthalmitis murine model attenuated the clinical inflammation, reduced the bacterial load in the retina, and preserved intact retinal architecture with normal electroretinogram (ERG) response. They also mentioned that intravitreal injection of Pam3Cys, alone or with antibiotics (vancomycin and ceftazidime) 24 h after *S. aureus* infection significantly improved the outcome of endophthalmitis in B6 mice (unpublished data).

In another similar study Kochan et al. [17] also demonstrated the benefit of the TLR2 ligand in a mouse model of *S. aureus *endophthalmitis. They studied the behaviour of retinal microglia, normally present in the inner and outer plexiform layers, and found that TLR2 expression in this population was increased and activated cells presence was frequent in the ganglionar cell layer of the infected animals. They also demonstrated that pre-endophthalmitis treatment with TLR2 ligand significantly increased their phagocytic activity and reduced the inflammatory response.

## 3. Cytokines

Cytokines are polypeptides that act as intercellular messengers that play an important role in mediating processes of inflammation and repair. They are secreted by macrophages, lymphocytes, natural killers, endothelial cells, in vitro RPE cells and other immune cells [19, 20]. The cytokines include tumour necrosis factors (TNFs), interleukins (ILs), interferons (IFNs), and a number of growth factors. They have been grouped into four categories or phases of inflammatory reaction.

- Recognition (mainly IL-1 and TNF-*α*); they are rapidly expressed and basic for the establishment of cytokine networks.
- Recruitment (called “chemokines”—human IL-8); they are essential for the elicitation of leukocytes.
- Removal (mainly IFN-*γ*, IL-2, and IL-6); their function is the activation of macrophages (IFN-*γ*) or lymphocytes (IL-2 and IL-6).
- Repair (growth factors); necessary to restore tissue structures.

The endophthalmitis immune response generates cell activation and cytokine secretion to suppress the infectious process. Petropoulos et al. [19] used an animal model of endophthalmitis caused by *S. epidermidis* to serially measure the levels of TNF-*α*, IL-1*β*, and IFN-*γ*. TNF-*α* and IL-1*β* behaved in a similar fashion, they peaked earlier, at 12 h after injection, while IFN-*γ* reached its maximum levels later, at 48 h. At day 7 after injection there was no statistically significant difference in cytokine levels between the experimental and the control groups. Clinical inflammation behaved in a similar way with the peak occurring slightly after the cytokines. TNF-*α*, IL-1*β*, and IFN-*γ* were not detected systemically, suggesting only local production. Clinical signs of endophthalmitis peaked at 24 h and by day 7 they were virtually nonexistent. TNF-*α* and IL-1, both produced by macrophages, are considered to be early initiators of this inflammatory process [16]. 

However it is not clear how the intraocular immune reaction starts. Rosenbaum showed in 2 studies that administration of a human IL-1 receptor antagonist (IL-1ra) did not block endotoxin-induced uveitis (EIU) in rabbits and that inhibitors of TNF-*α* also failed to block EIU [21, 22]. In another uveitis model, Brito et al. [23] demonstrated reverse passive Arthus reaction (RPAR), that mice deficient for the 2 known TNF-*α* receptor (TNFR) or IL1 receptor type I or both had a significantly reduced infiltration by inflammatory cells. The difference was the greatest in mice deficient for both receptors.

A specific endophthalmitis model in mice deficient for TNF-*α* with *B. cereus* resulted in reduced inflammation, more rapid bacterial replication, retinal function loss, and compensating proinflammatory cytokines. Chemokines were synthesized in the eye in the absence of TNF-*α*, resulting in less inflammation but an equally devastating course of infection [24].

High-mobility group box 1 (HMGB1) proteins are another class of molecules that have been identified in high concentrations in endophthalmitis and experimental uveitis [25, 26]. They are an abundant nonhistone nuclear protein with a dual function dependent upon its cellular location. In the nucleus, HMGB1 binds to DNA and is critical for proper transcriptional regulation. HMGB1 can also be passively released into the extracellular milieu by necrotic cells and secreted by activated macrophages, acting as a necrotic marker of tissue damage and a proinflammatory cytokine-like mediator.

Arimura et al. [25] studied the presence of HMGB1 in cases of human endophthalmitis using idiopathic macular holes as controls, and found that the HMGB1 levels in the vitreous were significantly elevated in eyes with endophthalmitis, especially those with a longer disease duration. Interestingly, HMGB1 concentration was significantly correlated with visual acuity; patients having the higher concentrations had lower visual acuity (VA). They also analysed one enucleated eye because of endophthalmitis secondary to corneal ulcer. HMGB1 was present in the cytoplasm and nuclear region of the choroid and retina, diffusely in all retinal layers including outer segments, outer plexiform layer, and especially in the damaged ganglion cell layer with infiltrating inflammatory cells. In a control enucleated eye due to a malignant conjunctival melanoma, HMGB1 was observed predominantly in the nuclei of retinal and choroidal cells suggesting that reduced HMGB1 concentration during endophthalmitis may reduce inflammatory induced retinal damage. Interestingly HMGB1 serum levels are elevated in sepsis, and an experimental sepsis model with specific inhibition of HMGB1 activity demonstrated an improvement in the clinical course and survival rate [27, 28].

## 4. Apoptosis 

Apoptosis, or programmed cell death for controlled deletion of unwanted cells, involves a sequence of events including blebbing, cell shrinkage, nuclear fragmentation, condensation of nuclear chromatin and DNA fragmentation. Finally apoptotic bodies are produced, and these are engulfed and quickly removed by phagocytic cells before the contents of the cell can spill out onto surrounding cells and cause damage. Apoptosis is controlled by a wide range of cell signals that may originate either intracellularly (intrinsic or mitochondrial pathway) or extracellularly (extrinsic pathway). Internal cellular damage upregulates the Bax protein which pricks the mitochondrial membrane forming high-conductance channels that allow release of cytochrome C from the mitochondria to the cytosol and activate caspases, which ultimately lead to cell death [29]. Extracellular signals may include toxins, hormones such as glucocorticoids, growth factors, nitric oxide, or cytokines [30]. When extrinsic aggression occurs, apoptosis can be directly initiated by TNF receptors or Fas receptors, both activating the caspase enzymes and leading to cell death.

In endophthalmitis, infection and inflammation involve many of these apoptosis-signaling molecules, and several experimental models have demonstrated that retinal cells apoptosis is increased [31, 32]. Pharmakakis et al. [31] created a model of *S. epidermidis *experimental endophthalmitis and found that there was an increased rate of apoptosis in correlation with upregulation of the expression of proapoptotic proteins Bax and Fas mainly within the ganglion cells, bipolar cells, and photoreceptors. Inflammation peaked at 24 hours after injection, Bax and Fas expression peaked at 48 hours after injection, and apoptotic rate peaked at 72 hours.

In a study to alter the normal apoptosis rate, Engelbert and Gilmore [32] tested the behaviour of FasL deficient mice in an *S. aureus *endophthalmitis model. They found that FasL expression on ocular tissues was essential for efficient clearance of* S. aureus.* Deficient FasL mice recruited less phagocytic cells and lost retinal function earlier with lower bacterial loads as compared with wild-type mice. Apoptosis through Fas is considered an important mechanism of maintenance of the immune privilege of the eye and contributes to the regression of the inflammatory process after the elimination of the etiologic factor [33].

aB-crystallin is a small heat shock protein that plays a critical role in protecting against apoptosis. It is expressed in long-lived tissues, such as muscle, brain and lens [34, 35]. It prevents apoptosis by inhibiting the activation of caspase 3 directly or indirectly by binding to the proapoptotic factors Bax and Bcl-X(S), by preventing their translocation into the mitochondria and by restricting the release of cytochrome C [36]. Recent studies have demonstrated that aB-crystallin is expressed in the retina (in the ganglion cells, inner and outer nuclear layers, inner segments and retinal pigment epithelium), where it is upregulated and prevents apoptosis in response to oxidative stress [37, 38]. Whiston et al. [39] analysed this protein in an *S. aureus *endophthalmitis model. They used wild and aB-crystallin knockout mice. Their results demonstrated that in the early response aB-crystallin is upregulated; however they also found that *S. aureus* produces a protease that cleaves and inactivates aB-crystallin. Deficient mice showed the same ability to clear the infection as wild mice but interestingly retinal function was significantly reduced and took more time to recover. Histological analysis demonstrated higher levels of apoptosis and retinal damage in the deficient mice.

## 5. Conclusions

Endophthalmitis often has a devastating effect on the eye and on visual function. Currently, vitrectomy, intravitreal antibiotics and corticosteroids are our main treatment options. Corticosteroids are the only available anti-inflammatory treatment used. The EVS advocated oral prednisone treatment (1 mg/Kg day) for 5 to 10 days, starting one day after intravitreal antibiotics [1]. More recently the use of intravitreal corticsteroids has been examined in several studies. Although they clearly seem to diminish the inflammatory reaction [40], their ultimate effect on the VA is contradictory as some studies have found a beneficial trend [41, 42], while others demonstrated no relation with the outcomes in eyes treated with intravitreal dexamethasone [43, 44]. Experimental endophthalmitis models have not clearly demonstrated the benefit of intravitreal dexametasone. Some studies have shown better electroretinogram function, less tissue destruction and reduced clinical inflammation scores in the groups treated with the combination of intravitreal antibiotics and dexametasone compared to intravitreal antibiotics alone [45, 46], while others demonstrated no benefit [47, 48]. Unfortunately, corticosteroids have not been demonstrated to successfully control the host immune reaction.

TLR2 ligand, Pam3Cys, has demonstrated encouraging results when administrated pre-endophthalmitis, but also when injected at 24 hours of the infection in combination with intravitreal antibiotics. In clinical practice, prevention of endophthalmitis is crucial; Pam3Cys properties are both prophylactic and therapeutic; if the results obtained by Kumar et al. [18] and Kochan et al. [17] are furtherly validated, a new treatment option that increases bacterial clearance and protects retinal function may be available. aB-crystallin upregulation during endophthalmitis seems to protect retinal functionality as it reduces apoptosis in retinal cells [39]; understanding better how this happens and managing to induce this upregulation can be an important step to achieve better functional results after endophthalmitis.

In order to improve the outcomes, it is essential the better understanding of the host immune reaction and the cellular pathways leading to tissue damage. Different types of research involving all the above mentioned mediators of inflammation are going on but we are still at initial stages. More efficient elimination of microorganisms, modulation of inflammation prior to retinal tissue damage and cellular protection are the pathways for the development of new therapeutic options that will help us to improve the final functional outcomes of this devastating condition.

## Figures and Tables

**Table 1:** Toll-like receptors and their known ligands.

TLR	Principal exogenous ligand(s)
TLR2	Lipoproteins/lipopeptides (various pathogens) Peptidoglycan and lipoteichoic acid (gram-positive bacteria) Zymosan (fungi)
TLR3	Double stranded RNA (viruses)
TLR4	LPS (gram-negative bacteria) Bacterial HSP6 Respiratory syncytial virus coat protein
TLR5	Flagellin (flagellated bacteria)
TLR7	Imidazoquinolone antiviral drug Double stranded RNA (viruses)
TLR8	Single stranded RNA (viruses) Imidazoquinolone antiviral drug
TLR9	Unmethylated cytidine-phosphate-guanosine CpG
TLR10	Unknown
